# BAKing up to Survive a Battle: Functional Dynamics of BAK1 in Plant Programmed Cell Death

**DOI:** 10.3389/fpls.2018.01913

**Published:** 2019-01-08

**Authors:** Xiquan Gao, Xinsen Ruan, Yali Sun, Xiue Wang, Baomin Feng

**Affiliations:** ^1^State Key Laboratory of Crop Genetics and Germplasm Enhancement, Nanjing Agricultural University, Nanjing, China; ^2^Jiangsu Collaborative Innovation Center for Modern Crop Production, Nanjing Agricultural University, Nanjing, China; ^3^Haixia Institute of Science and Technology, Fujian Agricultural and Forestry University, Fuzhou, China

**Keywords:** BAK1, development, co-receptor, programmed cell death, immunity

## Abstract

In plants, programmed cell death (PCD) has diverse, essential roles in vegetative and reproductive development, and in the responses to abiotic and biotic stresses. Despite the rapid progress in understanding the occurrence and functions of the diverse forms of PCD in plants, the signaling components and molecular mechanisms underlying the core PCD machinery remain a mystery. The roles of BAK1 (BRASSINOSTEROID INSENSITIVE 1-associated receptor kinase 1), an essential co-receptor of multiple receptor complexes, in the regulation of immunity and development- and defense-related PCD have been well characterized. However, the ways in which BAK1 functions in mediating PCD need to be further explored. In this review, different forms of PCD in both plants and mammals are discussed. Moreover, we mainly summarize recent advances in elucidating the functions and possible mechanisms of BAK1 in controlling diverse forms of PCD. We also highlight the involvement of post-translational modifications (PTMs) of multiple signaling component proteins in BAK1-mediated PCD.

## Introduction

Plants have evolved surveillance systems and cellular responses to sustain their growth while protecting themselves against various environmental stresses, often through deploying programmed cell death (PCD) to balance the survival signaling with proper development patterns and abiotic stresses or microbial infections (Van Hautegem et al., 2015; Kabbage et al., 2017). In response to microbial invasions, plants reply on cell surface receptor proteins to detect extracellular molecules produced by pathogens, collectively called pathogen-associated molecular patterns (PAMPs) and activate the PAMP-triggered immunity (PTI) pathway (Zipfel, 2014; Bigeard et al., 2015). Typical PTI responses include callose deposition, ROS production, and the expression of specific marker genes (Jones and Dangl, 2006). The receptor proteins located on plant cell surface that perceive PAMPs are known as pattern-recognition receptors (PRRs), which include receptor kinases (RKs) and receptor-like proteins (RLPs; Song et al., 1995; Gomez-Gomez and Boller, 2000; Chinchilla et al., 2006; Zipfel et al., 2006; Dardick et al., 2012).

In addition to PTI, a second layer of plant defense detects the presence of effector proteins from pathogens by intracellular immune receptor proteins, most of which are nucleotide-binding site leucine-rich repeat (NB-LRR or NLR) proteins and trigger robust immune responses, including ROS production, activation of specific effector-triggered immunity (ETI) marker genes, and rapid collapse of living tissues named as hypersensitive response (HR), a type of plant-specific PCD. PCD may limit the spread of pathogens; under other stresses, PCD may allow the recycling of nutrients to sustain growth (Jones and Dangl, 2006; Caplan et al., 2008; Eitas and Dangl, 2010; Feng and Zhou, 2012). In plant–microbe interactions, PCD has been recognized as a hallmark of ETI, and also important responses in certain PTI processes (Jones and Dangl, 2006; Caplan et al., 2008; Eitas and Dangl, 2010; Coll et al., 2011; Feng and Zhou, 2012). Other works revealed that PCD is a common and fundamental process that occurs in most eukaryotic organisms (Danon et al., 2000; Ameisen, 2002). PCD is also an intrinsic and indispensable process for plant vegetative and reproductive development. For example, differentiation and maturation of tracheary elements, and abscission of floral organs and tapetum degeneration all involve PCD. PCD was first reported in animals, where it serves as a mechanism to remove unwanted or damaged cells through development-related cell suicide and disease-related cell death (Zakeri et al., 1995; Raff, 1998).

Interestingly, plant PCD associated with development and immunity seems to be often connected to a cell-surface localized receptor kinase named BRI1-associated receptor kinase 1 (BAK1) (Chinchilla et al., 2007; Heese et al., 2007; Chinchilla et al., 2009; Ma et al., 2016). BAK1 was originally discovered as a key component of brassinosteroid (BR) signaling. Research in past decades demonstrated that BAK1 functions as a co-receptor in multifaceted receptor complexes to regulate a variety of processes, including BR-dependent development involving the receptor BRASSINOSTEROID INSENSITIVE 1-(BRI1), and Flagellin-Sensitive 2 (FLS2)-dependent PTI responses (Chinchilla et al., 2009; Ma et al., 2016). Readers who are interested in the topics about the role of BAK1 in innate immunity are strongly suggested to refer to several recent excellent reviews and herein (Ma et al., 2016; Yamada et al., 2016; Yasuda et al., 2017).

There is growing evidence to suggest that BAK1 plays an essential role in regulating various types of PCD (He et al., 2007; Kemmerling et al., 2007; Gao et al., 2009; Schwessinger et al., 2011; Gao et al., 2013b; de Oliveira et al., 2016; Du et al., 2016). The current review will focus primarily on the recent progress made in elucidating the functions of BAK1 in both development- and immunity-associated cell death. The possible mechanisms underlying BAK1-mediated cell death via its cooperation with multiple signaling components and its diverse regulatory mechanisms, including post-translational modification (PTM), will also be discussed.

## PCD in Plants

### Unique Features of Plant Cell Death

Based on the cell morphology, cell death in mammalian cells was classified into three types: apoptosis, autophagy, and necrosis (Zakeri et al., 1995; Kroemer et al., 2009; van Doorn, 2011). These three different types of cell death have different causes and different regulatory mechanisms. Apoptosis is characterized by early collapse and condensation of the nucleus, fragmentation of chromatin, generation of nucleosomal ladders, nuclear blebbing, and cytoplasmic condensation (Zakeri et al., 1995; Yu et al., 2002). The typical biochemical features of apoptosis include DNA cleavage, degradation of the DNA repair enzyme poly (ADP ribose) polymerase, and activation of caspases, a group of cysteine protease (Yu et al., 2002). Autophagy is normally characterized by an increase in the abundance of lytic vesicles termed autophagosomes (Berry and Baehrecke, 2008; Xu et al., 2015), and is regulated by a number of autophagy-related genes (*ATGs*) (Zhang and Tang, 2005; Patel et al., 2006; Berry and Baehrecke, 2008; Xu et al., 2015). Necrosis is typically accompanied by cell and organelle swelling, rupture of organelles and the plasma membrane, increases in ROS and calcium in cytoplasm, and decreases in cellular ATP (Farber, 1994; Postel et al., 2010; Heller et al., 2018). PCD is also involved in numerous biological processes in plants (Greenberg, 1996; Beers, 1997; Pennell and Lamb, 1997; Lam et al., 1999; Van Hautegem et al., 2015), such as cell differentiation and regulation of cell number, embryogenesis (Bozhkov et al., 2005), abiotic stress responses (Gepstein and Glick, 2013), and plant–pathogen interactions (Beers, 1997; Greenberg, 1997; Coll et al., 2011). Compared to animals, plants are sessile and plant cell surface is coated with much larger number of receptor proteins (∼600 RLK RLPs in Arabidopsis genome) (Tang et al., 2017), therefore, plant is thought to deploy differential, yet complexity strategies of PCD to adapt to diverse and harsh environmental stresses. To better understand the similarities and differences of PCD between plant and animal kingdoms, readers are suggested to refer to several excellent reviews (Kroemer et al., 2009; van Doorn, 2011; Rantong and Gunawardena, 2015; Dickman et al., 2017; Kabbage et al., 2017).

There are several commonalities of PCD features between plants and animals, including the existence of apoptosis-like morphological features and caspase-like proteases, as well as ATG genes in plants (Dickman et al., 2017; Kabbage et al., 2017). The hallmarks of PCD in mammalian systems, including the formation of apoptotic bodies and DNA cleavage (Mittler et al., 1995; Levine et al., 1996; Wang et al., 1996), also exist in plants (Greenberg, 1996). For example, in *Nicotiana benthamiana, BECLIN 1*, an ortholog of yeast ATG6/VPS30 and mammalian *beclin 1* has been identified and characterized as a key regulator in both developmental and HR-type PCD. *BECLIN 1* deficient *N. benthamiana* plants showed accelerated leaf senescence, while the HR-type cell death was suppressed in *BECLIN1*-silenced plants (Liu et al., 2005). However, many of the features and precise mechanisms of plant PCD are not fully understood.

Several features of PCD are distinct in plant cells, involving different types of caspases and phagocytosis systems compared with those in mammalian cells; the mechanisms involved in plant PCD differ from those in animal PCD, probably due to the genetic and functional redundancy of PCD components as well as plant-specific cellular features such as rigid cell walls, totipotency, and the presence of chloroplasts (Williams and Dickman, 2008).

The nature of apoptosis in plants is controversial. For instance, the viable aleurone cells of mature barley seeds undergo PCD when the seed starts to germinate, and the cells become highly vacuolated; however, aleurone cell death at this stage does not display the hallmarks of mammalian apoptosis-like PCD, probably due to the absence of major animal apoptosis regulators in plants (Fath et al., 2000; Dickman et al., 2017; Kabbage et al., 2017). Intriguingly, treatments with the Fumonisin B1 mycotoxin or abiotic stresses can trigger the formation of apoptosis-like bodies in plants (Li and Dickman, 2004; Li W. et al., 2010); therefore, caution should be paid when the presence of apoptosis-like cell death in plants is proposed (Dickman et al., 2017). Moreover, during senescence and the differentiation of the tracheary elements, cell death-associated physiological changes often involve vacuole collapse, providing evidence for the essential role of the vacuole in plant PCD (Jones, 2001). Besides vacuole, other organelles, including mitochondria and chloroplasts, have also been suggested to function in plant PCD (Lam et al., 2001).

Numerous environmental factors can trigger PCD, including salt (Li et al., 2007), drought (Duan et al., 2010; Hameed et al., 2013), ozone (Overmyer et al., 2005), and heat (Zuppini et al., 2007; Li Z. et al., 2012). When Arabidopsis roots were subjected to water deficit stress, for example, the typical features of PCD, including increased vacuole size, organelle degradation, and the collapse of tonoplast and plasma membrane, were observed in the apical meristem of the *Arabidopsis* primary root (Duan et al., 2010). Prolonged salt stress for 24 h caused by treatment with either NaCl or KCl resulted in the significant degradation of organelles in the green algae *Micrasterias denticulate* (Affenzeller et al., 2009). All together, these studies point to the existence and complexity of different forms of PCD in plants, in response to differential types of stresses. It has to be noted that, however, it remains enigmatic whether a common core machinery is shared for different types of PCD upon perception of either developmental or various abiotic or biotic stress cues (Huysmans et al., 2017).

### Classification of PCD in Plants

The classification of PCD in plants is contradictory, depending on the criteria. It was divided previously into two classes based on morphological features: vacuolar cell death and necrosis (van Doorn et al., 2011) but later updated to autolytic and non-autolytic cell death (van Doorn, 2011). Based on the triggers of PCD in plants, however, several previous studies have suggested that PCD could be classified into development-related PCD (dPCD), environment-related PCD (ePCD), and pathogen-triggered PCD (pPCD) (Daneva et al., 2016; Huysmans et al., 2017). dPCD is morphologically characterized by senescence, vacuolar collapse, nuclear degeneration or fragmentation, and cell elimination, which facilitates the successful establishment of reproductive organ identity and structural determination (Daneva et al., 2016). dPCD also occurs during vegetative development in plants, such as xylogenesis, as well as in organ abscission and dehiscence, where it is characterized by tonoplast rupture, vacuolar content release, mitochondrial degradation, and cytoplasmic clearance (Kuriyama, 1999; Yu et al., 2002). ePCD is thought to arise as a response to stress caused by diverse environmental conditions, including abiotic and biotic factors (Wu et al., 2014; Petrov et al., 2015). To better conceptually delineate the mechanisms involved in PCD, we propose that PCD is classified into dPCD, aPCD (abiotic stress-related PCD), and bPCD (biotic stress-related PCD). Currently, there is very limited information for the direct function of BAK1 in controlling aPCD; therefore, we will only focus on the involvement of BAK1 in the regulation of dPCD and bPCD in the following sections.

## BAK1 is Involved in the Regulation of Diverse Forms of PCD

BAK1 belongs to the SERK (somatic embryogenesis-related kinase) family, which are a small group of membrane-localized RLKs that can perceive diverse extracellular ligand stimuli and relay these signals, normally via a phosphorylation cascade (Li, 2010). The first plant SERK identified, *DcSERK*, was detected in carrot (*Daucus carota*) hypocotyl cell suspension cultures (Schmidt et al., 1997) during a search for marker genes to enable the monitoring of the transition from somatic cells into embryogenic cells. Most SERKs contain a small extracellular LRR-domain with five repeats, a single transmembrane domain, and a cytoplasmic kinase domain (Li, 2010). The *Arabidopsis thaliana* genome encodes five SERKs, *AtSERK1*, *AtSERK2*, *AtSERK3*, *AtSERK4*, and *AtSERK5*, which arose through gene duplication (Aan den Toorn et al., 2015). Baudino et al. (2001) isolated and identified maize (*Zea mays*) *ZmSERK1* and *ZmSERK2* using degenerate primers based on *DcSERK* and *AtSERK1*, and *ZmSERK3/BAK1* was later characterized for its function in embryogenesis (Zhang et al., 2011). Identification of SERK homologs in sequenced genomes of both higher plants and lower plants, such as moss (*Physcomitrella patens*), suggests an evolutionarily conserved significance of SERKs (Aan den Toorn et al., 2015).

To date, numerous genetic and biochemical studies have demonstrated that BAK1 functions as a master player at the convergence of multiple physiological processes, including the regulation of development, and responses to biotic stresses (Heese et al., 2007; Chinchilla et al., 2009; Schwessinger et al., 2011; Shen et al., 2011; Meng et al., 2015). For instance, BAK1 participates in BR signaling, vascular differentiation, stem elongation, flowering, floral abscission, fertility, and senescence (Li et al., 2002; Nam and Li, 2002; Postel et al., 2010; Meng et al., 2016). It was also reported to function in PHYTOSULFOKINE alpha (PSK)-regulated root growth (Ladwig et al., 2015), ERECTA (ER) and EPIDERMAL PATTERNING FACTORS (EPFs)-dependent cell fate specification in stomatal patterning (Meng et al., 2015).

How can BAK1 as a single RLK participate in so many different signaling pathways? One important reason is that BAK1 could function as a co-receptor or signaling regulator of multiple receptor kinases and RLPs; for example, BAK1 forms complexes with BRI1 to activate BR signaling, and with FLS2 to regulate a PTI pathway (Nam and Li, 2002; Chinchilla et al., 2007; Kemmerling et al., 2007; Ma et al., 2016). Additionally, BAK1 cooperates with multiple immune-related RLKs or RLPs at either the plasma membrane or in the cytoplasm, modulating distinct PCD processes (Table 1).

**Table 1:** Different forms of BAK1-mediated PCD in plants.

PCD type	Trigger of PCD	Receptor	Perturbation of BAK1 function	PCD phenotype	Possible Regulation	Reference
PCD by disruption of BAK1 and related proteins	*bkk1/serk4*		*bak1*	Spontaneous cell death	Phosphorylation; Glycosylation	He et al., 2007; Kemmerling et al., 2007; Jeong et al., 2010; de Oliveira et al., 2016
	BAK1 OE		BAK1 over-expression	Spontaneous cell death, BRI1 OE suppress this cell death		Domnguez-Ferreras et al., 2015; Kim et al., 2017
	SOBIR1 OE		NA	Spontaneous cell death	Phosphorylation	Gao et al., 2009; Liebrand et al., 2014
	*bir1*		*bak1*	Spontaneous cell death dependent on SOBIR1	Phosphorylation	Gao et al., 2009
	*bir2*		*bak1*	enhanced HP upon pathogen	Phosphorylation	Halter et al., 2014; Liu et al., 2016; Imkampe et al., 2017
	*bir3*		*bak1*	Enhanced HR	Phosphorylation	Halter et al., 2014; Imkampe et al., 2017
	*bon1*		NA	Spontaneous cell death	Phosphorylation	Wang et al., 2011; Kim et al., 2017;
	CRK28 OE		NbSerk3 silencing	Suppressed HR	Glycosylation	Yadeta et al., 2016, 2017
	*bir1pad4-1*		*sobir7-1/bak1*	Suppressed HR		Liu et al., 2016; Wu et al., 2018
bPCD by PAMPs	SCFE1 (Sclerotnia)	RLP30-SOBIR1	*bak1*	suppressed HR		Zhang et al., 2013
	NLP (bacteria, fungi, oomycete)	RLP23-SOBIR1-BAK1	*bak1*	suppressed HR		Albert et al., 2015
	INF1 (*Phytophthora infestans*)	Sl ELR-SOBIR1	*bak1*	Suppressed HR		Heese et al., 2007; Chaparro-Garcia et al., 2011; Domazakis et al., 2018
	BcXYG1 (*Botrytis*)	BAK1-SOBIR1	*bak1*	Suppressed HR		Zhu et al., 2017
	BcScp1 (*Botrytis*)		*bak1*	Suppressed HR		Dagvadorj et al., 2017
	Eix (fungal)	LeEix1/2 (LRR-RLP)	BAK1 silencing	Suppressed HR		Bar et al., 2010
	Pst SCR1 (*Puccinia stratiform*)		*bak1*	Suppressed HR		Dagvadorj et al., 2017
	pep2	PEPR	*bak1*	Enhanced HR		Yamada et al., 2016
bPCD upon pathogen	Hpa		*bak1*	Enhanced HR		Heese et al., 2007
	Alternaria		*bir2*	Enhanced HR		Halter et al., 2014
bPCD by effector	Aphid	Mi-1 (NLR)	*bak1*	Suppressed HR		Peng et al., 2016
	FolAvr1 (*Fusarium*)	I (LRR LRP)	*bak1*	Suppressed HR		Catanzariti et al., 2017
	Avr4/9 (Cladosporium)	Cf-4, Cf-9	*bak1*	Suppressed HR	Endocytosis	Postma et al., 2016
dPCD	TDIF	PXY	*bak1*	Treachery element PCD		Ma et al., 2016; Zhang et al., 2016
	TPD1	EMS1	*serk1/2*	Tapetal PCD		Li Z. et al., 2017
	IDA	HSA/HSL2	*serk1/2/3*	PCD in abscission zone		Meng et al., 2016


### BAK1 in Controlling dPCD

Numerous studies have revealed that BAK1 plays crucial roles in regulating dPCD; for example, silencing *GhBAK1* in cotton (*Gossypium hirsutum*) triggers high levels of cell death accompanied by increased ROS production, suggesting that the regulation of cell death by BAK1 is conserved in diverse plant species (Gao et al., 2013b). Interestingly, the BAK1 homolog, SERK5, does not regulate cell death in the *Arabidopsis* ecotype Col-0, whereas in the ecotype Landsberg *erecta* it has a regulatory role in cell death (Wu et al., 2015). The serine/threonine protein kinase BOTRYTIS-INDUCED KINASE 1 (BIK1) functions with BAK1; in the *bak1bik1* double mutant, a constitutive immune response and spontaneous cell death causes severe growth defects and a dwarf phenotype, accompanied with enhanced expression levels of immune genes, including *PR1*, *PR5*, *PAD4*, *WRKY45*, and *ERF1* (Liu et al., 2017). Additionally, BAK1 interacts with BIR1 (BAK1-interacting receptor-like kinase 1), and the *bir1* mutant displays a constitutive cell death phenotype (Gao et al., 2009). Both BAK1 and BIR1 interact *in vitro* and *in vivo* with BONZAI1 (BON1), a calcium-dependent phospholipid-binding protein; *bon1* mutants genetically interacted with *bir1* to produce temperature-dependent growth defects and cell death in *Arabidopsis* (Wang et al., 2011).

It has been noted that BAK1, together with other SERK family members, could function as a co-receptor of PXY (phloem intercalated with xylem) (Zhang et al., 2016). PXY is a LRR-RLK receptor of tracheary element differentiation inhibitory factor (TDIF), also known as CLAVATA3/EMBRYO SURROUNDING REGION-RELATED (CLE), which regulates vascular development in *Arabidopsis*, revealing the involvement of these essential components in dPCD (Ma et al., 2016; Zhang et al., 2016). It has been demonstrated that tracheary elements (TEs) typically undergo an autophagic type of PCD during differentiation in *Zinnia elegans* and *Arabidopsis* (Fukuda, 1997; Fukuda, 2000; Turner et al., 2007; Williams and Dickman, 2008). This type of PCD is characterized by a clearing process for the removal of dead protoplasts, normally achieved by multiple proteases, including xylem cysteine proteases 1 and 2 (XCP1 and XCP2), bifunctional nuclease 1/endonuclease 1 (BFN1/ENDO1) and metacaspase 9 (MC9) (Avci et al., 2008; Bollhoner et al., 2013; Xu et al., 2018). Some transcriptional factors, such as *VASCULAR-RELATED NAC-DOMAIN6/7 (VND6/7)*, appear to also be responsible for the PCD process activated by TDIF signaling (Ito and Fukuda, 2002; Pyo et al., 2007; Zhong et al., 2010; Heo et al., 2017). However, evidence supporting that BAK1 directly regulates dPCD is still missing.

Intriguingly, BR signaling was also found to be involved in xylem differentiation, as evidenced by the findings that treatment using the BR signaling inhibitor brassinazole, and the BR biosynthesis inhibitor uniconazole, resulted in aberrant vascular patterning and PCD (Yamamoto et al., 1997; Asami et al., 2000), while the BR deficient mutant, *cpd* (photomorphogenic dwarf) showed defective xylem biogenesis (Szekeres et al., 1996), and *bri1* single mutants and *bri1 brl1 brl3* triple mutants all displayed severe vascular defects (Cano-Delgado et al., 2004). Furthermore, upon perception of TDIF ligands CLV3 and CLE41, the TDIF receptor (TDR) interacts with BIN2 (Brassinosteroid-Insensitive 2), a member of GSK3 (Glycogen Synthase Kinase 3), to suppress procambial cell differentiation into xylem, which also involves the suppression of BES1 (BRI1-EMS Suppressor 1) downstream of TDR-GSK3 (Kondo et al., 2014; Heo et al., 2017). Given that BAK1 forms a signaling complex with BRI1 and PXY, respectively, it is possible that BAK1 acts as a convergent component shared by the PXY/TDR and BRI1 signaling pathways in dPCD regulation, yet this hypothesis remains to be further investigated and approved.

The BAK1 homologs SERK1 and SERK2 might also play a role in regulating dPCD in anther, in which they interact with the receptor-like kinase EMS1 to perceive the signal of a peptide ligand, TPD1, and control the differentiation of tapetum (Li Z. et al., 2017). Degeneration of tapetum through PCD and the release of its content to nurture maturation of pollen is essential for the success of male reproductive development. In *serk1, serk2*, or *ems1* mutants, tapetum differentiation and PCD were not properly initiated (Li Z. et al., 2017). This is a scenario similar to that in tracheary element differentiation. Again, a direct link between SERK1/2 and tapetal PCD needs solid experimental support. In the abscission zone, an IDA-HAS/HSL2 signaling pathway also relies on SERK members (SERK1/2/3) to transduce signals for abscission, where PCD is essential (Meng et al., 2016). Whether it is a common theme that BAK1 and other SERKs function upstream of certain dPCDs awaits future studies.

### BAK1 in Controlling bPCD

#### Functional Perturbation of BAK1 and Its Partners Triggers bPCD

BAK1 functions as a co-receptor of multiple RLKs and is involved in diverse signaling pathways. BAK1’s function seems essential and is under tight surveillance so that PCD is triggered once its function is perturbed. Previously, a genetic investigation on the single mutation of BAK1 itself and double mutation of BAK1 with its closest homolog BKK1 (SERK4) revealed that while *bak1* showed strong premature senescence (Kemmerling et al., 2007; Jeong et al., 2010), cell death in *bak1 bkk1* double mutants occurs post-embryogenesis, suggesting that BAK1 and BKK1 function redundantly to negatively control cell death (He et al., 2007; de Oliveira et al., 2016). *bak1* single mutants developed a type of uncontained PCDs upon infection with virulent necrotrophic pathogens, which differs from both necrotizing elicitor- and SA-inducible PCD (Kemmerling et al., 2007). Similarly, silencing BAK1 in *N. benthamiana* also leads to enhanced PCD upon infection with *Hyaloperonospora parasitica* (Heese et al., 2007).

Interestingly, over-expression of BAK1 or its ectodomain also elicited spontaneous PCD, accumulation of SA and expression of multiple PCD-related genes, including *BON1*, *BIRs*, and *SOBIR1* (Kim et al., 2017). In line with this finding, constitutive expression of BAK1 or its ectodomain or excess of BAK1 could trigger strong dwarfism and premature death phenotype, as well as autoimmunity without microbe attacks (Domnguez-Ferreras et al., 2015). Therefore, the abundance of BAK1 seems important and needs to be kept in check. It is hypothesized that over-expression of BAK1 might sequestrate BIR1 to trigger PCD (Ma et al., 2016). At present, it remains unclear whether *bak1*/*bkk1* cell death is due to the loss of negative regulation of PCD by BAK1 or is caused by an unknown mechanism that monitors developmental defects in *bak1*.

Multiple signaling components distinct from the BRI1 pathway are also engaged by BAK1 to trigger PCD upon pathogen infection. For instance, a BAK1-interacting RLK, BIR1, was identified by a reverse genetics approach; the *bir1-1* mutant displayed extensive cell death and constitutive immunity (Gao et al., 2009). Intriguingly, further suppressor screening using *bir1-1* led to the identification of *suppressor of bir1-1* (*sobir1-1*), which strongly suppressed the cell death observed in *bir1-1* (Gao et al., 2009). Moreover, over-expression of *SOBIR1* results in elevated cell death, indicating that SOBIR1 functions as a positive regulator of cell death (Gao et al., 2009; Liebrand et al., 2014). Using Co-IP coupled with liquid chromatography-electrospray ionization-tandem mass spectrometry (LC/ESI-MS/MS), two close BIR1 homologs, BIR2 and BIR3, were identified and demonstrated to constitutively interact with, and be phosphorylated by, BAK1, which in turn prevents the formation of the BAK1–FLS2 receptor kinase complex, thus negatively regulating PTI signaling (Halter et al., 2014; Imkampe et al., 2017). Examination of an allelic series of *bak1* mutation showed that BIR1 and BAK1 interact genetically to regulate BR signaling, cell death and immune response (Wierzba and Tax, 2016).

*bir1* has enhanced SA-dependent PCD (Liu et al., 2016). Moreover, upon infection with a necrotrophic pathogen, *Alternaria brassicicola*, *bir2* mutants had enhanced cell death and susceptibility to this pathogen, whereas BIR2 interacts with BAK1 and suppresses the autoimmune cell death response in the absence of PAMPs (Halter et al., 2014). Furthermore, it was reported that while BIR3 interferes with BRI1-dependent growth by interacting with and stabilizing BAK1, it also negatively affects the formation of the BAK1–FLS2 complex to suppress cell death and immunity, exemplified by the enhanced spontaneous cell death in the *bak1 bir3* mutant (Imkampe et al., 2017).

#### BAK1 Is Involved in Certain PAMP-Triggered PCD

As a co-receptor of multiple RLKs that perceive PAMPs, BAK1 is required for certain PAMP-triggered PCD; for example, in *N. benthamiana*, triggering of ROS accumulation and HR by the INF1 protein secreted from the oomycete pathogen *Phytophthora infestans* was prevented by a mutation in BAK1 (Heese et al., 2007; Chaparro-Garcia et al., 2011). A recent study showed that silencing of *SOBIR1* in *N. benthamiana* attenuated INF1-triggered cell death and resistance to *P. infestans*, while SOBIR1 was found to form a receptor complex with ELR (Elicitin Response) protein isolated from *Solanum microdontum*, which is a RLP perceiving INF1 (Domazakis et al., 2018). Moreover, BAK1 is recruited to the ELR/SOBIR1 signaling complex to activate downstream defense response, suggesting that both SOBIR1 and BAK1 are required for INF1-regulated PCD and immunity. Similarly, BcXYG1, a xyloglucanase protein secreted from *Botrytis cinerea*, interacts with BAK1 and SOBIR1 to trigger cell death and the immune response (Zhu et al., 2017). A small apoplast-targeted cysteine-rich protein, PstSCR1, secreted from the wheat rust pathogen *Puccinia striiformis f. sp. tritici*, triggers PCD and immunity in *N. benthamiana* via a pathway that appears to be dependent on the BAK1 pathway (Dagvadorj et al., 2017).

Moreover, BAK1 cooperates with SOBIR1 and RLP30 (Receptor-Like Protein 30) to control PCD caused by necrotrophic pathogens. RLP30 is responsible for the sensitivity to SCLEROTINIA CULTURE FILTRATE ELICITOR1 (SCFE1)-containing fraction, which contains a proteinaceous elicitor, produced by *S. sclerotiorum*, and *rlp30* mutants showed increased cell death and susceptibility to infection with necrotrophic pathogens *S. sclerotiorum* and *B. cinerea* (Zhang et al., 2013). In addition, BAK1 was found to interact with one of the LRR-RLP receptors in *N. benthamiana*, LeEix1, to attenuate the LeEix2-mediated Eix (Ethylene-inducing xylanase) response in tobacco (*Nicotiana tabacum*) and tomato (*Solanum lycopersicum*) in order to trigger the typical HR response (Bar et al., 2010).

Using proteomic approaches several RLKs, cysteine-rich receptor-like kinases (CRKs) enriched at the plasma membrane, were identified while the expression levels of these *CRKs* were activated upon the ligand elicitation of flagellin in *Arabidopsis* (Yadeta et al., 2017). Among those CRKs, the induction of CRK28 activity was highly correlated with enhanced resistance to the wheat rust pathogen *P. striiformis f. sp. tritici* and increased ROS production; moreover, the kinase active site of CRK28 (K377) is required for triggering cell death. CRK28 associates with the FLS2/BAK1 immune complex in a flg22-dependent manner, and CRK28-induced cell death was abolished in *NbSerk3*-silenced *N. benthamiana* plants, suggesting that BAK1 is required for CRK28-mediated cell death (Yadeta et al., 2017).

However, in some cases, loss of BAK1 function enhances the cell death triggered by PAMPs or DAMPs. A recent study showed that when PTI signaling is compromised by BAK1 disruption, danger peptide receptor PEPRs (Pep Receptors) signaling could be activated to ensure basal resistance. Depletion of BAK1 sensitized PEPRs signaling toward cell death upon ligand elicitation, as the ligand Pep2 was found to induce extensive cell death in *bak1-4* mutants, which is dependent on PEPRs (Yamada et al., 2016). It is believed that such an enhanced PCD phenotype in *bak1* mutant upon pathogen infection is caused by the subsequent dysfunction of BAK1/BON1 suppressed cell death, which in turn activates the PEPR signaling pathway to reversely trigger cell death and to retain immunity to biotrophic pathogens (Yamada et al., 2016).

#### BAK1 and Effector Triggered PCD

Lines of evidence suggest that PCD triggered by perturbation of BAK1 functions shows similarity to R-protein-mediated PCD. SALICYLIC ACID INDUCTION-DEFICIENT (SID2) and ENHANCED DISEASE SUSCEPTIBILITY5 (EDS5), two chloroplast-localized components of the salicylic acid (SA)-mediated ETI pathway, were also proposed to contribute to cell death by BAK1/BKK1 mutations, thereby regulating PCD; the *sid2* and *eds5* mutations suppress cell death in *bak1-3bkk1-1* mutants, and this cell death is dependent on light and SA (Gao et al., 2017). In line with this finding, the over-expression of BAK1 resulted in the accumulation of SA and hydrogen peroxide, as well as the enhanced expression of *BON1*, *BIRs*, and *SOBIR*, the processes strongly associated with spontaneous cell death (Kim et al., 2017). BON1, functioning as a negative regulator of R-mediated resistance, interacts with both BAK1 and BIR1 to interfere with the immune response and PCD (Wang et al., 2011). Furthermore, enhanced cell death in *bir1* plants was found to be partially dependent on PHYTOALEXIN DEFICIENT4 (PAD4) and EDS1, which is required for TIR NLR signaling. This suggests that BIR1 might be guarded by plant resistance (R) protein signaling (Gao et al., 2009).

BAK1 seems also to participate in other PCDs during ETI. Two effectors from the tomato pathogen *Cladosporium fulvum*, Avr4 and Avr9, are recognized by the R proteins Cf-4 and Cf-9, respectively, which in turn recruit BAK1 and subsequently trigger the HR and immunity against *C. fulvum* (Postma et al., 2016). The BAK1/SOBIR1-dependent pathway was also shown to mediate the interaction between the tomato resistance gene I, a LRR-RLP involved in the immunity to *Fusarium oxysporum* f. sp. *lycopersici* (*Fol*), and the *Fol*Avr1 effector, triggering necrosis in *N. benthamiana* (Catanzariti et al., 2017). Moreover, BAK1 positively regulates the NLR protein Mi-1 in resistance and cell death upon potato aphid infection in the tomato (Peng and Kaloshian, 2014; Peng et al., 2016).

## Possible Mechanisms Underlying Contradictory Function of BAK1 in PCD

As reviewed above, BAK1 is involved in different forms of PCDs. However, it is intriguing that BAK1 could serve as both positive and negative regulators of PCDs. The question about how BAK1, as a single RLK, is capable of controlling different PCD processes oppositely remains open to answer. Here, we would like to summarize the possible molecular mechanisms that might explain the complicated function of BAK1 in PCD.

It is possible that the output specificity of BAK1 functions in different PCD processes is determined by the ligand specificity of corresponding RLKs. Therefore, the effects of disrupted BAK1 functioning are likely dependent on the role of BAK1 in that specific complex. BAK1 also interacts with other RLKs including BIR1, BIR2, and SOBIR1. The balance between different BAK1-incorporated complexes is obviously influenced by the abundance of BAK1. These interacting RLKs might keep each other under tight control to ensure proper activation of PCDs. It has been shown that both BIR1 and BIR2 appear to be essential in structurally keeping BAK1-regulated PCD under tight control in the case of no ligand binding, thus interfering the unwanted interaction between BAK1 and different PRRs (Ma et al., 2017). Moreover, a specific ligand stimulates the release of BIR2-sequestered BAK1, which subsequently enhances the interaction complex formation between BAK1 and PRR, FLS2, EFR, BRI1, PEPR1, etc., (Halter et al., 2014). Interestingly, BIR1, BIR3, and BIR4 all formed a stable heterodimeric complex with BAK1 at pH 6.0 through their ecto-domains, and flg22-bound FLS2 outcompeted BIR1^LRR^ for binding to BAK1^LRR^ (Ma et al., 2017). On the other hand, upon pathogen infection, *bak1bir3* showed increased pathogen-inducible PCD (Imkampe et al., 2017); upon ligand perception, overexpression of BIR2 suppressed the BAK1/FLS2 (PRR) complex formation (Halter et al., 2014). Moreover, BAK1 overexpression results in runaway cell death, and simultaneous overexpression of BRI1 and BAK1 suppresses this cell death, suggesting that activation of the BAK1–PRR signaling complex upon ligand binding is essential to suppress this type of auto-immune PCD (Belkhadir et al., 2012; Halter et al., 2014).

The downstream signaling events upon activation of PRR–BAK1 complexes also show specificity to ligands. A very recent study suggests that the specificity might be determined by the phosphosite code in BAK1. They identified multiple BAK1 phosphosites specific to the signaling processes, e.g., immunity or growth, but not others (Perraki et al., 2018). In this case, the mutation of multiple key phosphorylation sites in BAK1, including S602D/T603D/S604D or S612D, resulted in impaired PTI responses, including flg22-induced MAPK signaling and immunity to *Pst* DC3000, but dispensable for BR-signaling. Moreover, the mutation of Y403 in the BAK1 C-terminal attenuated the phosphorylation and BAK/EFR signaling complex formation upon elf18 elicitation (Perraki et al., 2018). Other studies also support that the intracellular domains have separable functions in mediating different signaling processes. In a *bir1* suppressor screen using *bir1-1pad4-1* mutant, *sobir7-1*, a *bak1* allele with a nonsense mutation within the carboxyl-terminal tail (CT) of BAK1, was identified (Liu et al., 2016; Wu et al., 2018). A series of genetic evidence proved that the CT domain of BAK1 was essential for its kinase activity to trigger PTI response, but dispensable for controlling cell death and BR signaling (Liu et al., 2016; Wu et al., 2018). It is worth investigating how the differential regulation of PCD involving BAK1 might also attribute to the diverse phosphosite codes activated.

As discussed above, the PCD triggered by disruption of BAK1 might be mediated by certain R proteins, which is a well-accepted theme that host protein is guarded by cognate R proteins. If this is true, the cell death caused by BAK1/BKK1 loss-of-function does not imply they are negative regulators of PCD, but instead that they are such important signaling components that their disruption is monitored by R-protein. Some cases whereby R proteins guard important host signaling components have been reported. For example, RIN4 is guarded by two NLRs, RPM1 and RPS2, and disruption of the MAP kinase cascade, MEKK1/MKK5/MPK4, will trigger the activation of SUMM2 R-protein and cell death (Zhang et al., 2012). Moreover, PAD4-dependent immunity is activated in *bir1-1*, raising the possibility that BIR1 is guarded by R gene, too (Liu et al., 2016). Indeed, it has been proposed that BAK1/BIR1 is probably guarded by two or more R proteins in the absence of pathogens. Mutation of BAK1 or BIR1 results in the activation of those guard R proteins, which subsequently trigger different signaling pathways, e.g., disease resistance and/or PCD, mediated by PAD4 and SOBIR1, respectively, (Gao et al., 2009). In line with this finding, SRF3, an LRR-RLK structurally similar to BIR2, has been reported to regulate hybrid incompatibility along with the R gene *RPP1*, during which necrotic PCD, enhanced SA levels and immune response were found. This illustrates a model that SRF3 is guarded by RPP1 to control incompatibility in the absence of pathogens (Alcazar et al., 2010).

Nevertheless, as a center component involved in various signaling pathways, it appears that an optimal amount of BAK1 should be strictly maintained to optimize the fitness of growth/development and disease resistance/PCD. While being tightly guarded by and released from R proteins, and responding to appropriate ligands to interact selectively with different PRRs, BAK1 has evolved multiple strategies to cooperate with diverse signaling components to fine-tune its function in regulating different types of PCD.

## Regulation of BAK1-Mediated PCD

Several studies have suggested that the perception of PAMPs by the PRRs and the subsequent activation of downstream signaling are associated with multiple regulation events, especially PTMs (such as phosphorylation, ubiquitination, glycosylation), and protein endocytosis (Lu et al., 2011; Kadota et al., 2014; Lin et al., 2014). The importance of some of these processes has also been demonstrated to be associated with the functional dynamics of BAK1-mediated PCD (Bender et al., 2015; de Oliveira et al., 2016; Table 1).

As discussed above, BAK1 signaling specificity might be determined by the code of phosphosites (Perraki et al., 2018). The phosphorylation of non-RD plasma membrane-localized LRR-RKs, including FLS2 and EFR, by BAK1 is essential for inducing PTI upon the perception of PAMPs, which is different from that of RD-kinase BRI1 phosphorylation by BAK1 (Schulze et al., 2010; Schwessinger et al., 2011). In this case, the *bak1-5* mutant, a novel mutant *BAK1* allele carrying a single amino acid substitution, C408Y, in the BAK1 cytoplasmic kinase domain, is impaired in PTI signaling but not in cell death regulation. Upon ligand elicitation, however, BAK1-5 kinase activity is required for the formation of BAK1/FLS2 or BAK1/EFR PRR complexes. Moreover, the *bak1-5* line becomes insensitive to SCFE1, and did not show enhanced PCD. This was different from *bak1-3* and *bak1-4* mutants, which are susceptible to *B. cinere*a and *A. brassicicola* (Kemmerling et al., 2007). These findings strongly support the mechanistically uncoupled and phosphorylation-dependent activation function for BAK1 in regulating distinct signaling pathways, e.g., BR-associated development, PCD and PTI responses (Schwessinger et al., 2011; Monaghan and Zipfel, 2012).

BAK1 is also capable of phosphorylating BIK1 at tyrosine and serine/threonine sites, as evidenced by the requirement for kinase activity and the presence of three tyrosine residues (Y150, Y243, and Y250) in BIK1 for its function in immunity (Lin et al., 2014). Furthermore, upon PAMP elicitation, BIK1 could directly interact with and phosphorylate RBOHD to control the ROS burst and promote resistance to bacterial pathogens (Kadota et al., 2014).

In addition to phosphorylation, other PTM processes participate in the regulation of BAK1 activity. For instance, the PAMP flg22 triggers the recruitment of a pair of plant U-box E3 ligases, PUB12 and PUB13, to FLS2, which is subsequently degraded by ubiquitination. Interestingly, FLS2 ubiquitination by PUB12/13 requires the BAK1-mediated phosphorylation of FLS2 (Lu et al., 2011). Since it has been shown that disruption of *PUB13* caused a spontaneous cell death phenotype, which was also enhanced under high humidity conditions (Li W. et al., 2012), it is reasonable to speculate that an ubiquitination event might also be involved in BAK1-mediated PCD.

Using the BAK1 cytoplasmic domain as bait to screen a yeast two-hybrid library, a glutaredoxin (GRX) C2 (AtGRXC2) protein was characterized to be a BAK1-interacting component. AtGRXC2 can *S*-glutathionylate and form a heterodimer with the BAK1 cytoplasmic domain *in vitro* in the presence of either glutathione disulfide or glutathione plus H_2_O_2_, thus inhibiting BAK1 kinase activity (Bender et al., 2015). BAK1 kinase activity was enhanced by the mutation of an AtGRXC2-targeted essential glutathionylation site, Cys408 to tyrosine, in a *bak1-5* background (Bender et al., 2015). Although it remains to be determined whether BAK1 glutathionylation is directly associated with its role in the regulation of PCD and immunity, this finding reveals a novel regulatory mechanism of BAK1 signaling by redox status and glutathionylation.

The process of endoplasmic reticulum (ER)-mediated protein quality control (ERQC) and glycosylation were shown to be important for BAK1-mediated PCD as well. To identify the suppressor of cell death that silences BAK1/SERK4 (BKK1) and BIR1, a virus-induced gene silencing (VIGS)-based genetic screen was carried out in the *bak1-4serk4-1* and *bir1* mutant backgrounds, leading to the identification of a mutant with a defective STAUROSPORIN AND TEMPERATURE SENSITIVE3 (STT3a) protein (de Oliveira et al., 2016). The *stt3a-2* mutation significantly suppresses cell death, H_2_O_2_ accumulation, and *PR1* and *PR2* expression in the *bak1-4serk4-1* and *bir1* backgrounds, providing strong genetic evidence for the positive role of STT3a in triggering PCD and the immune response (de Oliveira et al., 2016). Interestingly, several specific ERQC components, such as ERdj3b and SDF2, seem to be involved in triggering cell death in the *bak1-4/serk4-1* mutants (Sun et al., 2014). On the other hand, using an RNA-seq analysis, one of the most highly activated gene families in *bak1-4*/*serk4-1* was found to be the CRKs, including *CRK4* and *CRK5*, which strongly elicit cell death when transiently overexpressed in *N. benthamiana* (de Oliveira et al., 2016). Moreover, a biochemical analysis showed that CRK4 and CRK5 are likely the targets of glycosylation, as revealed by an obvious migration shift under electrophoresis. Given the known function of STT3 as a catalytic subunit of oligosaccharyltransferase in protein *N*-glycosylation, these findings suggest that STT3a-mediated *N*-glycosylation and ERQC are essential for CRK4-mediated PCD. It remains to be determined whether CRK4 and CRK5 are necessary and sufficient for the cell death in *bak1-4 serk4-1* mutant plants (de Oliveira et al., 2016). This result is further supported by the finding that CRK28 is also a glycosylated transmembrane protein found in a PRR–RLK complex (Yadeta et al., 2017). Intriguingly, it is noted that a subset of LRR-RK-type PRRs, including EFR and Xa21, specifically require this ERQC pathway in their proper folding and maturation (Saijo, 2010; Beck et al., 2012), implying the possible recruitment of a similar LRR-RK to mediate cell death in the absence of BAK1.

In addition to the aforementioned ERQC pathway and glycosylation, nucleocytoplasmic trafficking is also essential to BAK1-mediated PCD. *sbb1-1*, another suppressor of cell death in *bak1-4 serk4-1*, was identified in a genetic screen (Du et al., 2016). *SBB1* encodes a nucleoporin (NUP) 85-like protein that is a member of the NUP107-160 sub-complex, the largest sub-complex known to be highly conserved in vertebrates and plants. Knocking out individual NUP members including *SBB1* (*NUP85*), *SEH1*, *NUP160*, or *NUP96* fully suppresses the cell death phenotype of the *bak1-4* and *serk4-1* mutants. The *sbb1* mutation reduced endogenous SA levels and the *sbb1* mutant suppressed cell death in *bak1-4* and *serk4-1*, and expression of *SBB1* driven by its own promoter in *bak1-3 bkk1-1 sbb1-2* can recapitulate cell death phenotype, suggesting that SBB1-mediated cell death in *bak1-4serk4-1* is SA-dependent (Du et al., 2016). Interestingly, co-immunoprecipitation coupled with LC-MS/MS analyses identified numerous SBB1-interacting proteins, including DEAD-box RNA helicase 1 (DRH1), which was found to directly associate with SBB1. Genetic data demonstrated that SBB1-DRH1 is required for cell death in *bak1-4* and *serk4-1*. Consistent with the observation that DRH1 is localized at the nucleus and that SBB1 functions in mRNA export, the SBB1–DRH1 complex-mediated nucleocytoplasmic trafficking process likely contributes to BAK1/SERK4-controlled cell death, which might be exerted through its interference in the export of SA-related mRNAs (Du et al., 2016).

Endocytosis is also involved in BAK1-mediated PCD. For example, interaction of BAK1 with LeEix1 results in the endocytosis of LeEix2, whereas LeEix1 interferes with the LeEix2-triggered immune response and HR, which was impaired in the BAK1-silenced plants (Bar et al., 2010). This finding reveals the key role of BAK1 in regulating Eix-induced PCD and the PTM of the LRR-RLP receptor upon pathogen infection.

## Conclusion and Perspectives

Cell death is an essential process for both mammals and plants. Despite the remarkable progress made in the elucidation of the occurrence and features of PCD, more research is needed to determine how host plants perceive and transduce external signals to activate PCD. Similar to animals, PCD is deployed by plants to facilitate cell differentiation during development or to promote survival by enabling plants to adapt to environmental stresses and defend against pathogens. Accumulating evidence points to the central role of BAK1 as a co-receptor or signaling regulator of multiple receptor kinases and RLPs in different types of PCD. BAK1 likely exerts its function via deploying its specific phosphorylation sites to phosphorylate PCD-related RLKs or RLPs (Perraki et al., 2018), differentiating responses to the elicitation by various ligand bindings, and to distinctly modulate PCD.

BAK1 has been intensively investigated for its function as the key component of the BR-mediated signaling pathway and the RLK-mediated PTI signaling pathway; thus, it is reasonable to theorize that dPCD (meristem cell death) and bPCD converge at BAK1. It has been proposed that calcium signaling (for example, via the CDPKs) (Boursiac et al., 2010; Gao et al., 2013a) and ROS production are involved in the regulation of both dPCD and bPCD (Gechev and Hille, 2005; Boursiac et al., 2010; Petrov et al., 2015; Serrano et al., 2015). Given the pivotal role of BAK1 as a co-receptor of the PTI signaling complex in triggering PCD, it is tempting to speculate that BAK1 may function upstream of ROS and/or calcium signaling to regulate the diverse types of PCD, probably through the activation of a MAPK signaling cascade (He et al., 2007; Kemmerling et al., 2007; Jeworutzki et al., 2010; Gao et al., 2013b).

PCD is considered to be a hallmark of the ETI response. Upon ETI activation, PCD can also be triggered via CDPK-mediated signaling, likely through the phosphorylation of specific WRKY transcription factors (Gao et al., 2013a); however, it is unclear whether BAK1 exerts its negative role in controlling PCD as either a shared core regulator of ETI-triggered PCD or through distinct mechanisms. There is accumulating evidence to suggest that BAK1 could be targeted by multiple effectors (Macho and Zipfel, 2014); for instance, by AvrPtoB (Shan et al., 2008) and HopF2 (Zhou et al., 2014) from *Pseudomonas syringae*, and Avr3a from *P. infestans* (Chaparro-Garcia et al., 2011), resulting in disruption of PTI signaling as well as that of PCD suppressed by BAK1. Furthermore, SOBIR1 associates with or interacts with several R proteins (Qi et al., 2011; Ma and Borhan, 2015); thus, it is possible that activation of ETI-associated PCD might be attributed, at least to some extent, to the negative regulation by BAK1 and BAK1/SOBIR1 receptor complexes. One should be cautious, however, that BAK1 targeting by different effectors leading to enhanced cell death may not be pertinent to all forms of BAK1. This is because BAK1-5 is a hypoactive kinase and the *bak1-5* mutant is not impaired in cell death control (Schwessinger et al., 2011), whereas the kinase domain of BAK1 seems to be essential for AvrPtoB targeting structurally (Cheng et al., 2011).

PCD functions at essential steps in development and aging as well as in abiotic and biotic stresses. It seems that plants may deploy the BAK1 signaling complex to coordinate different types of PCD and thus control the trade-off between development and immunity, possibly via subverting hormone signaling, interacting with R proteins, and integrating distinct PTM processes. Future research should center on exploring how host plants control PCD by orchestrating BAK1 homeostasis, which may also provide practical implications for crop improvement. Moreover, despite the fact that animal apoptosis-like cell death has not been fully addressed in plants, recent evidence has suggested the presence and functional importance of animal-type apoptosis in plant PCD (Dickman et al., 2017). Another research direction will therefore involve clarification of the relationship between BAK1-regulated PCD and apoptosis-like cell death, as well as other cell death processes, such as autophagy, under different stresses or environmental stimuli.

## Author Contributions

XG wrote the manuscript. XG and BF revised the manuscript with input from XR, YS and XW.

## Conflict of Interest Statement

The authors declare that the research was conducted in the absence of any commercial or financial relationships that could be construed as a potential conflict of interest.
